# The dynamic impact behavior of the human neurocranium

**DOI:** 10.1038/s41598-021-90322-3

**Published:** 2021-05-31

**Authors:** Johann Zwirner, Benjamin Ondruschka, Mario Scholze, Joshua Workman, Ashvin Thambyah, Niels Hammer

**Affiliations:** 1 Department of Anatomy, University of Otago, Dunedin, New Zealand; 2 Institute of Legal Medicine, University Medical Center Hamburg-Eppendorf, Hamburg, Germany; 3 Institute of Legal Medicine, University of Leipzig, Leipzig, Germany; 4 Institute of Materials Science and Engineering, Chemnitz University of Technology, Chemnitz, Germany; 5 Institute of Macroscopic and Clinical Anatomy, Medical University of Graz, Graz, Austria; 6 Department of Chemical and Materials Engineering, University of Auckland, Auckland, New Zealand; 7 Department of Orthopedic and Trauma Surgery, University of Leipzig, Leipzig, Germany; 8 Fraunhofer IWU, Dresden, Germany

**Keywords:** Musculoskeletal system, Bone, Biomedical engineering

## Abstract

Realistic biomechanical models of the human head should accurately reflect the mechanical properties of all neurocranial bones. Previous studies predominantly focused on static testing setups, males, restricted age ranges and scarcely investigated the temporal area. This given study determined the biomechanical properties of 64 human neurocranial samples (age range of 3 weeks to 94 years) using testing velocities of 2.5, 3.0 and 3.5 m/s in a three-point bending setup. Maximum forces were higher with increasing testing velocities (*p* ≤ 0.031) but bending strengths only revealed insignificant increases (*p* ≥ 0.052). The maximum force positively correlated with the sample thickness (*p* ≤ 0.012 at 2.0 m/s and 3.0 m/s) and bending strength negatively correlated with both age (*p* ≤ 0.041) and sample thickness (*p* ≤ 0.036). All parameters were independent of sex (*p* ≥ 0.120) apart from a higher bending strength of females (*p* = 0.040) for the 3.5 -m/s group. All parameters were independent of the post mortem interval (*p* ≥ 0.061). This study provides novel insights into the dynamic mechanical properties of distinct neurocranial bones over an age range spanning almost one century. It is concluded that the former are age-, site- and thickness-dependent, whereas sex dependence needs further investigation.

## Introduction

Biomechanical parameters characterizing the load-deformation behavior of the human neurocranium are crucial for building physical models^1–3^ and high-quality computational simulations^4–6^ of the human head to answer complex biomechanical research questions to the best possible extent. Human head models are utilized to simulate various impact scenarios such as falls^7^, vehicle accidents^8^, gunshots injuries^9^ or forensically relevant accident reconstructions in court. These scenarios are of a dynamic, especially a non-quasi-static, nature, which requires the biomechanical properties of a viscoelastic material such as bone^10^ to be obtained using appropriate experimental setups. Such setups then allow for an encompassing material description resulting in lifelike physical or computational replications of bone. Previous studies predominantly focused on the load resistance of the human neurocranium using static testing velocities^1,11–18^, while only a few studies applied dynamic testing velocities^19,20^. The anatomical complexity of the human neurocranium, especially the mechanically relevant site-dependent thickness differences^14,21^ of the three-layered bone composite multiplies the required demands in testing for an accurate mechanical description. Mechanical characterizations of the individual neurocranial bones so far mainly focused on the frontal^1,12–20,22^ and parietal^1,12–20,22^ areas. Despite its clinical importance in fractures due to lateral head impacts like vehicle accidents^23^, the temporal bone remained sparsely investigated using dynamic testing speeds to date^22^. When considering head impacts, the neurocranium is of particular importance as it protects the human brain as a vital organ. A recent study comparing the frontal, temporal, parietal and occipital bones when loaded to failure, showed that the occipital bone yielded the highest failure forces, whereas the temporal bones revealed the lowest^24^. With regards to elasticity, the frontal bone was the most and the temporal bone the least elastic with the parietal bone between the two^14,18^. Generally, the availability of cadaveric material for mechanical testing purposes is limited, restricting the concurrent investigation of demographic influences such as age (at death) and sex (of the deceased) on the mechanical parameters. Previous studies on the dynamic mechanical properties of the human neurocranium predominantly described either infants^17,19,25^ or the elderly^20^. This, in consequence, does not allow for general conclusions on the age-related mechanical properties of neurocranial bones over the entire human lifespan. Furthermore, dynamically obtained mechanical properties of the female neurocranium are sparse. It remains unclear to date whether sex has to be considered an independent variable of neurocranial failure loads. The relationship between sex and load resistance is to be carefully investigated as the human neurocranium reveals sex-dependent morphological differences that might be reflected in the mechanical properties as it was shown for other human tissues before^26^. The given study aimed to determine the load resistance of different human neurocranial samples when exerted to dynamic strain rates, while simultaneously assessing the influence of age, sex and thickness on the obtained mechanical properties. Special consideration was given to the load resistance of the temporal bone, which had been largely under-investigated in former studies.

## Material/methods

### Tissue retrieval and processing

**Figure 1:**
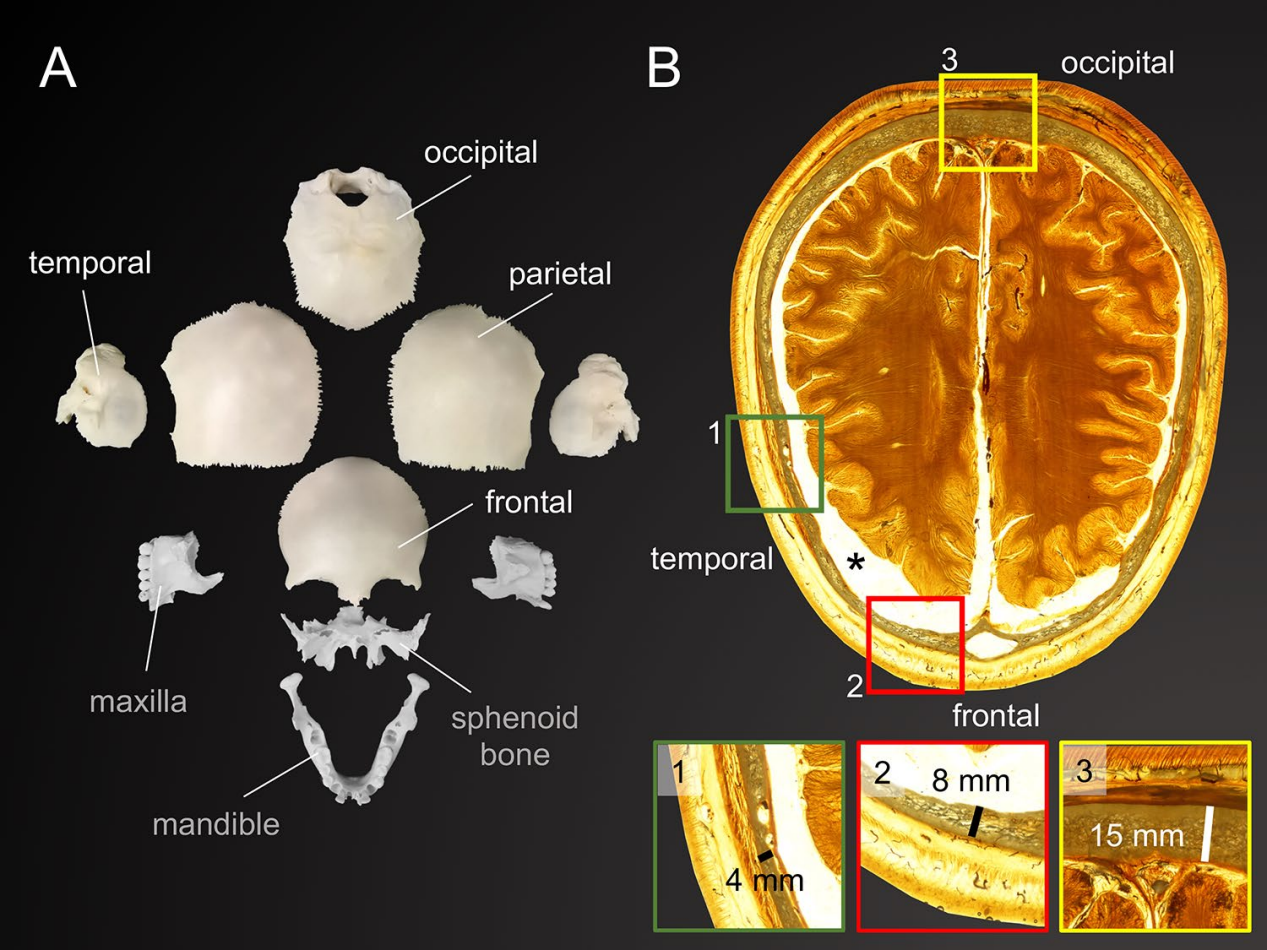
Two items of the museum collection of the Department of Anatomy, University of Otago are shown to illustrate the cadaveric material used in this study. (**A**) The individual bones of an infant human neurocranium are depicted. The four bones used for mechanical tests are labelled in white, additional bones are labelled with grey color. (**B**) Crosado-embalmed and subsequently E12-plastinated cross-sections (horizontal plane) of a human adult head illustrates the varying thicknesses of the neurocranium in various regions. The asterisk (*) indicates a shrinkage-related artefact in the subdural space, which is caused by the dehydration and degreasing steps of both the Crosado embalming^27^ and the E12 plastination^28^.

A total of 68 human skull samples were retrieved from 47 cadavers (16♀, 31♂; age range: 3 weeks to 94 years, mean: 48 years, median: 49 years) during forensic autopsies at a German Institute of Legal Medicine. The samples were retrieved from the frontal (n = 9), temporal left (n = 21), temporal right (n = 22), parietal (n = 7) and occipital (n = 9) region (Fig. 1) and were further processed without attached soft tissues such as dura mater or periosteum. The median postmortem interval (PMI), which refers to the time between the death of the cadaver and the autopsy, was 70 h with an interquartile range (IQR) of 34 h. Prior to the autopsy, the cadavers were stored at 4 °C at the earliest possible time post mortem to slow down tissue degradation. After retrieval, the samples were precooled and kept in a freezer at - 80 °C until further processing. All methods were carried out in accordance with relevant guidelines and regulations. Ethical approval was obtained from the Ethics Committee of the University of Leipzig, Germany (protocol number 486/16-ek). The samples were cut into beams with a uniform width of 10 mm using an ultrasonic bone cutter (PIEZOSURGERY white, mectron s.p.a., Carasco, Italy). The width of the samples was determined using a digital caliper (Coolant Proof 200 mm, MeasumaX, Auckland, New Zealand; accuracy ± 0.001″). The samples were allocated into three groups according to the different testing velocities of 2.5 (21 samples in total, 14 temporal), 3.0 (21 samples in total, 14 temporal) and 3.5 m/s (22 samples in total, 15 temporal), which were used for the impact tests in this study. The samples of the frontal, parietal and occipital areas were allocated to the three different groups.

### Mechanical testing

A custom-built drop tower^29^ was used for the dynamic mechanical tests in this study. The setup consisted of a semi-circular high-strength aluminium indenter with a radius of 1.5 mm that was fixed to a weighted cross-head (total weight including indenter = 643 g), which was attached to two vertically oriented stainless steel rods using polytetrafluoroethylene bushings. The indenter was connected to a piezoelectric force transducer (Model 9021A, Kistler AG, Winterthur, Switzerland) with the signal being fed to a charge amplifier (Model 5015A, Kistler AG) and recorded with an oscilloscope (TDS 360, Tektronix, Beaverton, OR, USA) using a sampling rate of 200 MHz. The bone samples were placed on two support beams with a radius of 2 mm. The support beams were 13 mm separated allowing for the indenter to impact the sample at the midpoint between the two beams following a three-point bending setup. The tests were recorded with a high-speed camera (2000 fps; i-speed 2HG, Olympus NDT Inc. Waltham, MA, USA) fitted with a macro lens and an LED light source. The indenter was released from three different heights allowing the indenter to hit the sample with impact velocities of 2.5, 3.0 and 3.5 m/s, corresponding to low fall heights of 32, 46 and 62 cm. The testing velocities at impact were optically verified using the Photron Fastcam Analysis software (Photron, Tokyo, Japan). The thickness of each sample was measured optically along a line that crossed the lengthwise midpoint of the tested sample (identical with the impact point of the indenter) perpendicular to the same using the Measure 2.1d software (DatInf, Tübingen, Germany).

### Data processing and statistical analyses

Bending strengths (B_strength_) and maximum forces (F_max_) were calculated from the aforementioned tests. For statistical evaluations Excel Version 16.16 (Microsoft Corporation, Redmond, WA) and GraphPad Prism software version 8 (GraphPad Software, La Jolla, CA) were used. The D’Agostino & Pearson test was used to test the Gaussian distribution of the samples. Following this, an unpaired one-tailed t-test was performed for comparisons with normally distributed datasets and an unpaired one-tailed Mann–Whitney test for datasets with a non-normal distribution, respectively. For comparisons involving more than two datasets, normally distributed data of samples were then tested using an ordinary one-way ANOVA (with post hoc Fisher’s LSD test for normally distributed data) or a Kruskal–Wallis test (with post hoc uncorrected Dunn’s test for non-normally distributed data), respectively. Bivariate correlations (Pearson’s r for normally distributed, Spearman’s r for non-normally distributed data) were conducted between the obtained mechanical parameters and age, PMI, sex and thickness of the samples. Medians and IQRs are given in the text. The comparisons were made for all samples as well as for the temporal samples individually (called ‘temporal only’ group). P values of 0.05 or less were considered statistically significant.

## Results

### Maximum force was significantly higher at increasing testing velocities while bending strength increased on a statistically non-significant level only

**Figure 2:**
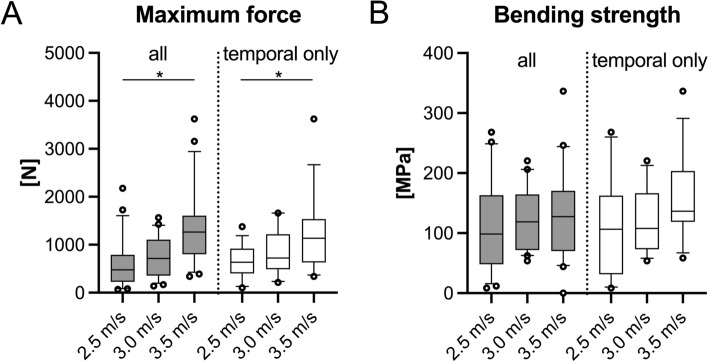
The maximum force (**A**) and bending strength (**B**) are depicted for the three different testing velocities for all samples (grey color) and temporal samples only (white color). Whiskers are defined as 10th and 90th percentile, circles represent outliers. *p ≤ 0.05.

The 2.5-m/s group resisted significantly lower F_max_ (716 N, IQR = 844 N) compared to the 3.5-m/s group (1264 N, IQR = 807 N, *p* = 0.031, Fig. 2A). The same was true when only temporal bone samples were compared (2.5 m/s: 638 N, IQR = 519 N, 3.5 m/s: 1136 N, IQR = 902 N; *p* = 0.026, Fig. 2A). Temporal bone samples of the 2.5-m/s and the 3.0-m/s group (722 N, IQR = 727 N) were significantly less resilient compared to the samples from other regions of the respective groups (2.5 m/s: 1543 N, IQR = 2377 N, *p* = 0.015; 3.0 m/s: 1859 N, IQR = 673 N, *p* = 0.009). The B_strength_ was similar and statistically non-different between all tested samples and regions (2.5 m/s: 98 MPa, IQR = 115 MPa; 3.0 m/s: 119 MPa, IQR = 92 MPa; 3.5 m/s: 130 MPa, IQR = 140 MPa; *p* ≥ 0.285). When the temporal bone samples were compared independent from the other bone samples, similar findings were observed (2.5 m/s: 107 MPa, IQR = 131 MPa; 3.0 m/s: 109 MPa, IQR = 93 MPa; 3.5 m/s: 137 MPa, IQR = 84 MPa; *p* ≥ 0.087, Fig. 2B).

### Correlations between mechanical parameters and specimen thickness, age at death, sex of the deceased and postmortem interval

**Figure 3:**
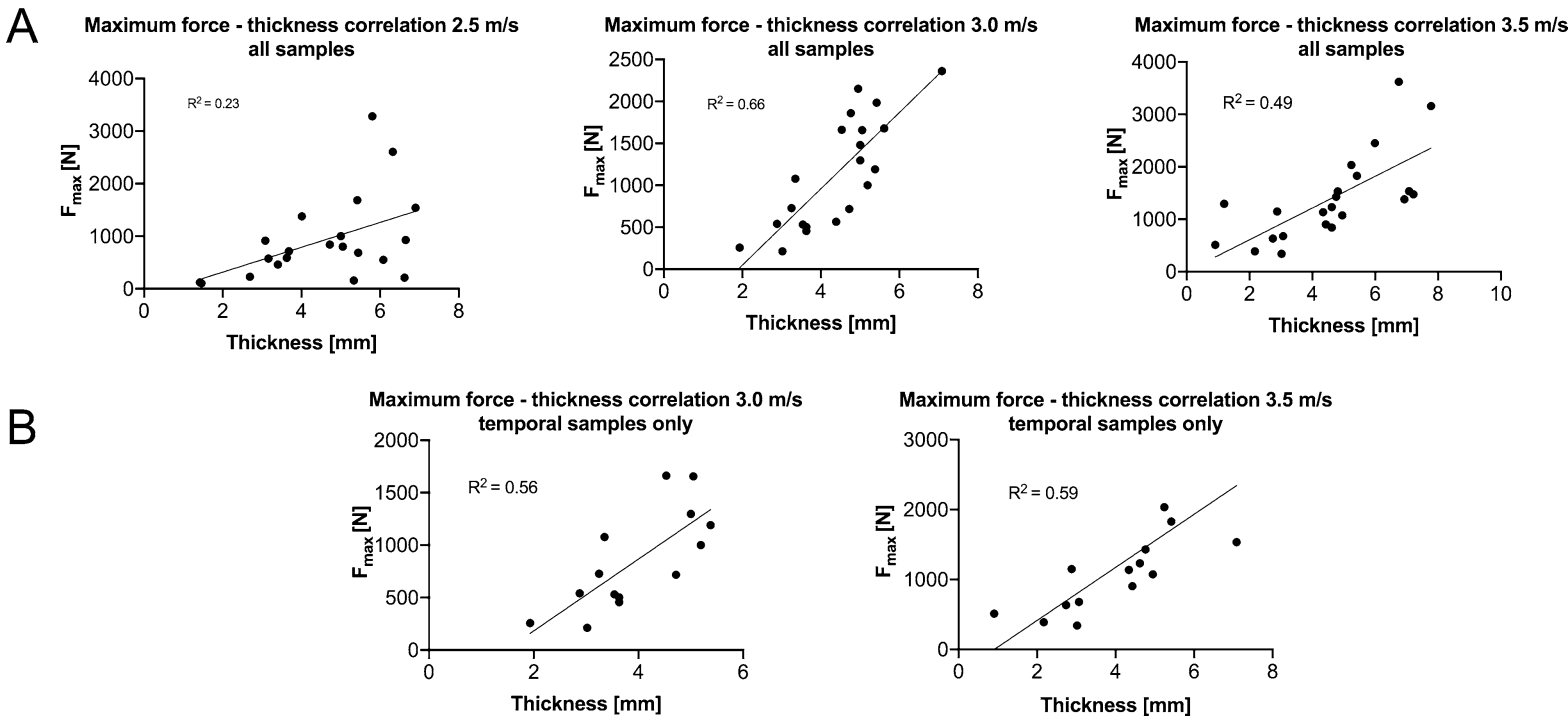
Statistically significant correlations between the maximum force (F_max_) and thickness are depicted for (**A**) all samples and (**B**) temporal samples only.

**Figure 4:**
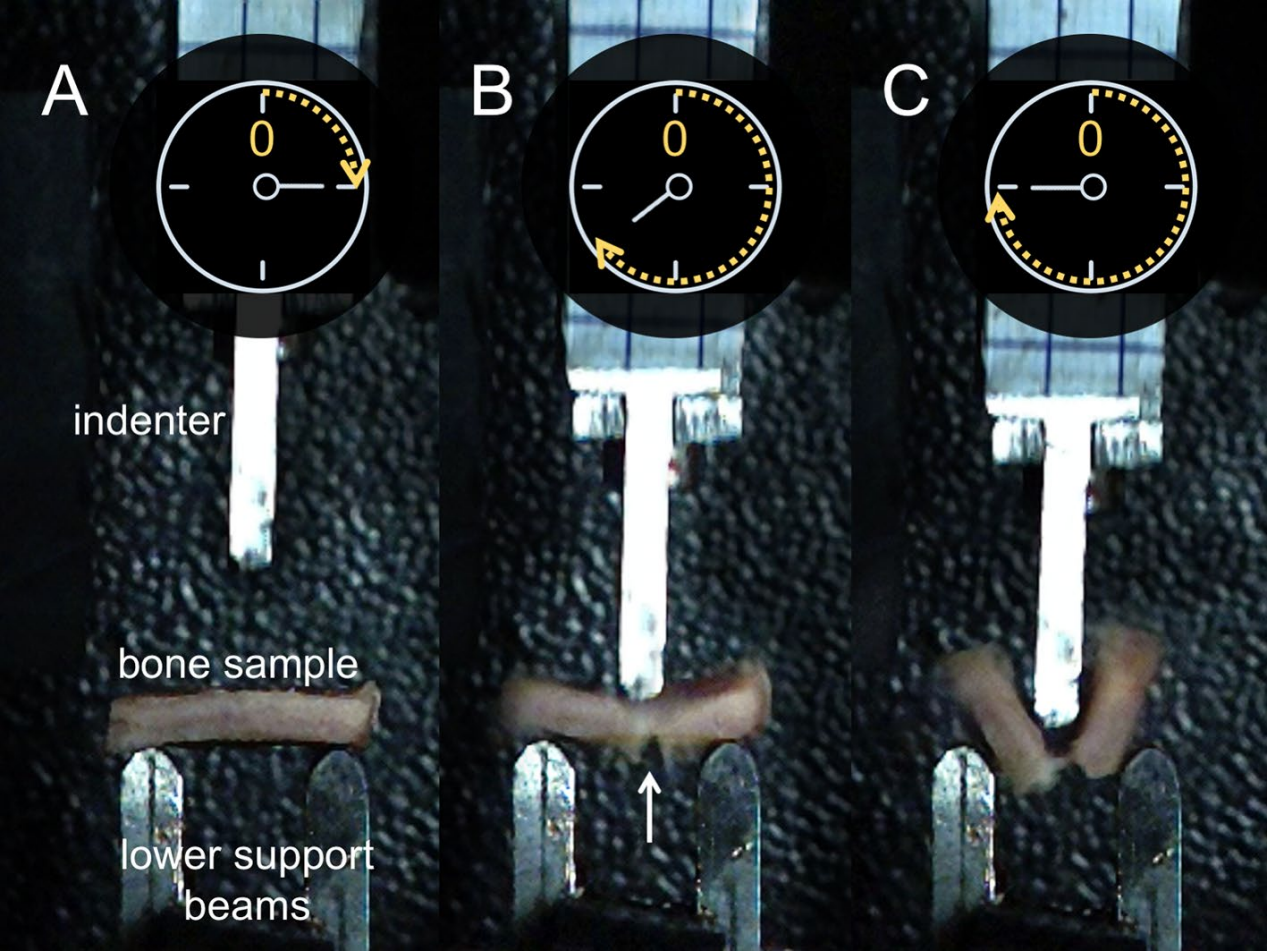
A drop tower test of a right temporal bone sample taken from a 23-year-old male is shown (**A**) before the indenter impacts the sample, (**B**) at impact, after the maximum force (F_max_) was reached with the fracture being clearly visible on the inner side of the bone (white arrow), and (**C**) with the entire bone being fractured. The sample was positioned according to a three-point bending setup. The two lower support beams with a separation distance of 13 mm were placed in a way ensuring that the indenter impacted the sample in the middle of the separation distance.

**Figure 5:**
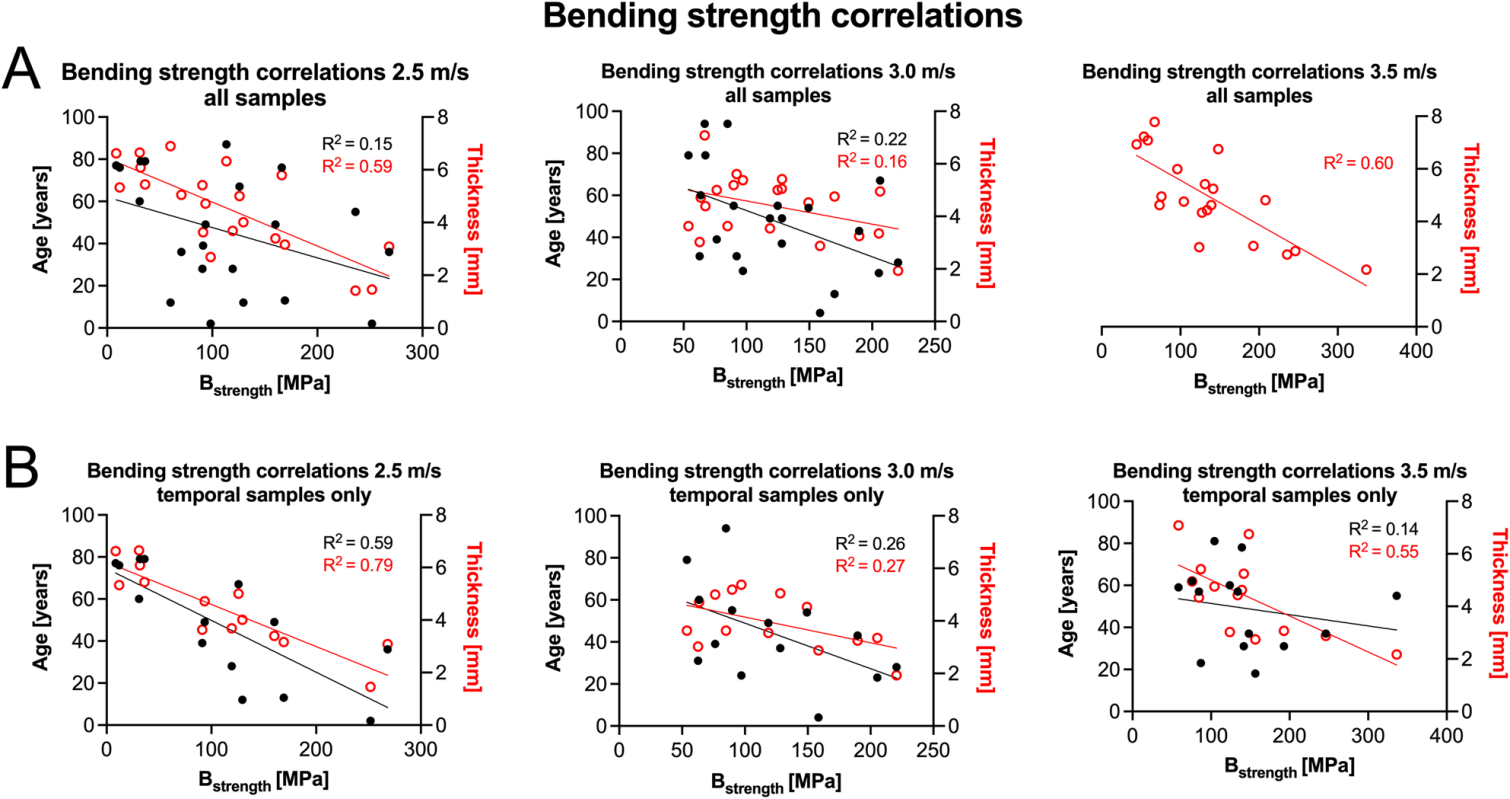
Correlation plots between the bending strength (B_strength_) and both age (black color, left y-axis) and thickness (red color, right y-axis) are depicted for (**A**) all samples and (**B**) temporal bone samples only, illustrating overall negative correlations between the variables.

Sample thicknesses strongly correlated positively with F_max_ of each individual group (2.5 m/s: r = 0.501, *p* = 0.012; 3.0 m/s: r = 0.812, *p* < 0.001; 3.5 m/s: r = 0.796, *p* < 0.001; Fig. 3A). When only temporal samples were considered, the thicknesses correlated positively in the 3.0 m/s (r = 0.747, *p* = 0.001) and 3.5 m/s (r = 0.850, *p* < 0.001) but not in the 2.5-m/s group (r = 0.085, *p* = 0.386; Fig. 3B). Representative video-captured images of the conducted drop tower tests are shown in Fig. 4. The B_strength_ negatively correlated with age for 2.5 m/s (r = − 0.389, *p* = 0.041) and 3.0 m/s (r = − 0.470, *p* = 0.016), but not for the highest testing velocity of 3.5 m/s (r = − 0.257, *p* = 0.137, Fig. 5A**)**. However, when only temporal samples were evaluated the B_strength_ negatively correlated with age irrespective of the applied testing velocity (2.5 m/s: r = − 0.767, *p* < 0.001; 3.0 m/s: r = − 0.512, *p* = 0.031; 3.5 m/s: r = − 0.631, *p* = 0.009; Fig. 5B). Moreover, the B_strength_ correlated negatively with the thickness of the samples in each velocity group 2.5-m/s: r = − 0.766, *p* < 0.001; 3.0 m/s: r = − 0.399, *p* = 0.036; 3.5 m/s: r = − 0.774, *p* < 0.001, Fig. 5A), which was consistent with the findings when the temporal group was assessed exclusively (2.5 m/s: r = − 0.889, *p* < 0.001; 3.0 m/s: r = − 0.520, *p* = 0.028; 3.5 m/s: r = − 0.591, *p* = 0.014 (Fig. 5B). Negative correlations between B_strength_ and age at death were observed for all but the 2.5-m/s group (*p* = 0.080) when only samples older than 18 years (as adulthood representatives) were investigated to exclude development-related influences (*p* ≤ 0.043). Also, the thickness was negatively correlated with the B_strength_ irrespective of the testing velocity when only samples of 18 years or older were evaluated (*p* ≤ 0.046). Females had a significantly higher B_strength_ (98 MPa, IQR = 69 MPa) compared to males (69 MPa, IQR = 47 MPa, *p* = 0.040) for the 3.5-m/s group of all tested samples. None of the other mechanical parameters was sex- (overall: *p* ≥ 0.072; ‘temporal only’: *p* ≥ 0.120) nor PMI-dependent (overall: *p* ≥ 0.072; ‘temporal only’: *p* ≥ 0.061).

### Consistency analysis of tested groups

The individual testing velocity groups were statistically non-different with regards to age (*p* > 0.546), PMI (*p* > 0.371) and specimen thickness (*p* > 0.700). When only temporal bone was analyzed, the velocity groups were non-different regarding age (*p* > 0.614), PMI (*p* > 0.723) and specimen thickness (*p* > 0.355). The pooled temporal samples (4.34 mm, IQR = 2.11) were thinner compared to the pooled other samples (5.42 mm, IQR = 1.92, *p* = 0.046). The thickness of all samples significantly increased with age (r = 0.421, *p* < 0.001), which also applied to the temporal bones only (r = 0.425, *p* = 0.002), and when samples older than 18 years were evaluated exclusively (r = 0.271, *p* = 0.023). There was no significant difference of sample thicknesses between the two sexes (r = 0.009, *p* = 0.471).

## Discussion

Human cadaveric material continues to be the preferred source for investigations determining the biomechanical behavior of the human neurocranium, thereby obtaining fundamental data for head injury criteria^23^. Only a few studies so far investigated the force tolerance limits of the human neurocranium under dynamic loading conditions^17,19,20,22^. This lack of study is partly related to tissue availability, and partly also to the experimental setup required to obtain meaningful and reproducible data. Fifty years ago, the dynamic tensile properties of 30 human neurocrania were determined on the frontal, temporal and parietal area of cadavers aged between 15 and 95 years using testing velocities between 0.0001 and 3.8 m/s^22^. No site dependency of the mechanical parameters was observed between the tested regions of the neurocranium, with only nine tested temporal specimens in total^22^. In contrast, the results of this given study revealed a significantly lower F_max_ of the temporal bone compared to the frontal and parietal areas. The data in this given study was obtained utilizing a three-point bending setup, which is the most frequently used setup for the mechanical characterization of bones^1,12,14–16,18,20^. Therefore, the tensile data obtained in the aforementioned study^22^ cannot be directly compared to other recent studies. Another study tested frontal and parietal bone samples of eight human crania aged between 62 and 97 years using dynamic testing velocities between 0.5 and 2.5 m/s in a three-point bending setup comparable to the study presented here^20^. The observed averaged F_max_ for the frontal, left parietal and right parietal bones using the highest testing velocity of 2.5 m/s were 1316 N, 1227 N and 1162 N^20^, respectively, which seems much higher compared to the here reported 716 N for all samples using an identical testing velocity. This may be explained by the following two facts: firstly, the here given study predominantly consisted of temporal samples, which were significantly thinner compared to the pooled frontal, parietal and occipital samples. This thickness difference is in line with previous studies on the thickness of the various regions of the neurocranium^16^. The quantity of diploë in temporal bone is low when compared to the other flat bones of the human neurocranium. It may even be nonexistent in particular areas^30^. The authors here hypothesize that diploë thickness appears to be of major interest for failure mechanisms of the human neurocranium as the results of the here given dynamic study and a previous quasi-static study^24^ revealed the lowest F_max_ values for temporal bone samples when compared to the major neurocranial flat bones. Secondly, the mean age of the cadavers of 48 years in the here given study was much lower compared to the 81 years of the aforementioned study^20^. A study that investigated the fracture loads of 94 cadavers in a static testing setup using a testing velocity of 0.0003 m/s reported mean F_max_ values between 435 and 515 N for temporal bone^16^. The here presented temporal F_max_ values were 638, 722 and 1136 N using testing velocities of 2.5, 3.0 and 3.5 m/s, respectively, being well in line with the former observations and showing an increasing trend with increasing velocities. Even though no statistical comparison can be provided between the two studies, the highest here used testing velocity of 3.5 m/s resulted in higher F_max_ values for the temporal samples compared to the values provided in the aforementioned study using a static testing setup^16^. This can likely be explained by the time-dependent, viscoelastic and strain rate-dependent character of osseous tissue^10^. However, it has to be noted that this given study and the one by Torimitsu et al.^16^ have technical differences including statistical considerations of the non-normally distributed F_max_ values being compared with mean values^16^ and the different distances of the lower supports with 13 and 50 mm. These impede the direct comparison of the data between the two studies. Also, temporal samples in this given study were thinner compared to the samples used in the referred static experiment with a median of 4.3 mm compared to a mean of 5.5 to 6.6 mm^16^.

This study highlighted the strong correlations between F_max_ and sample thickness, the here stated findings can be considered well in line with previous investigations after all, but provide a much broader age span and tissues were obtained from all major neurocranial bones. The age-dependent thickness increase of bone shown in this study might, even after adolescence, contribute to the increased failure loads in studies conducted on elderly people. This highlights that human head models have to be adjusted for an increasing thickness with increasing age as this has direct implications for the biomechanical behavior of the respective neurocranial bones. Supported by the here presented results, the thickness of the cranial sample seems the most predictive parameter to influence the applicable F_max_ of the individual sample^24,31^, which further supports the necessity of modelling realistic thicknesses for all bone parts of the neurocranium in both physical and computational head models. A study applying dynamic three-point bending tests (testing velocities between 1.2 and 2.8 m/s) conducted on fetus and infant neurocrania revealed that bending modulus and ultimate stress were independent of the applied strain rate^19^. In contrast to these findings, dynamic studies on adult tissues showed that increasing loading rates led to significantly higher elastic moduli, maximum bending stresses, F_max_^20^ and breaking strains^22^. The results of this study support the increase of F_max_ with increased loading rates, although a statistically significant result was only observed between the lowest and the highest testing velocity. Similarly, in the study by Motherway et al. a significant increase in F_max_ was only observed between testing velocities of 2.5 m/s vs. 1 and 0.5 m/s, respectively^20^. The high inter-individual scatter of mechanical parameters such as F_max_ might require larger sample sizes than presented here and formerly^20^ to detect minute differences in F_max_ at lower testing velocities or much higher velocity differences between the groups. The testing velocities in this given study are in line with head impacts in contact sports, including elbow to head impact (1.7 to 4.6 m/s) or head to head impacts (1.5 to 3.0 m/s) in football^32^. It has to be acknowledged that a variety of head impacts, including falls from moving bikes (approximately 11 m/s)^33^ or gunshot wounds, where the ammunition moves at a velocity of several hundred meters^34^ are far beyond the here tested velocity range. However, the authors hypothesize that the velocity range of head impacts in vivo covers the entire range from almost static to the highest velocity gunshot impacts.

Age-related biomechanical data of human tissues are of increasing interest, as the population is ageing, and both males and females are known to suffer from hormone-induced changes in bone metabolism associated with reduced mechanical bone strength related to osteoporosis^35,36^. Contrary to femoral neck or vertebral fractures, which are associated with an age-related decrease in bone density^37^, the age-associated fracture characteristics of the neurocranium are less obvious. This might be due to the lower frequency of injuries in this region overall. Some studies concluded that the neurocranium does not show a particular age-related decrease of mechanical strength^23^, whereas others reported a decrease of fracture loads with age^16^. To the authors best knowledge this is the first study to investigate dynamic biomechanical properties of the human neurocranium over the expected human life span of more than nine decades. F_max_ was unrelated to the age at death, which contrasts findings of another study that observed an age-dependent decrease of F_max_ applying static testing velocities^16^. B_strength_ decreased consistently with age in all testing velocities, which was present in both the overall group and the temporal group when considered independently. This may partly be explained by the increasing thickness of the neurocranium with age observed here, which is inversely proportional to the squared thickness of the bone sample^38^ according to the B_strength_ equation^12^. Failure stress, failure strain as well as energy absorption of human neurocranial samples remained constant with increasing age in a dynamic tensile setup^22^. The values F_max_, B_strength_ and thickness were independent of sex in the here investigated samples except for the significantly higher B_strength_ of females compared to males in the highest here tested velocity. Also, the F_max_ of occipital and parietal samples were shown to be higher for males in a static testing setup^16^. However, both observations should be investigated in a larger sample size to confirm these sex-related differences as the general trend appears to lean towards sex-independence of the biomechanical properties of the human neurocranium. Noteworthy, mechanical properties of female neurocranial bone samples are scarcely investigated in general^17,20^, and studies so far frequently investigated an uneven ratio of males compared to females^1,16,22^. This uneven ratio might partially be explained by the overall higher number of males compared to females in forensic autopsies^39^. Forensic autopsies are an important source to retrieve chemically and pathologically unaltered material for mechanical characterizations of human tissues in all age ranges^13,40–43^. This is in contrast to the oftentimes chemically altered anatomical dissections or pathologically altered clinical post mortems. The inclusion of sufficient female samples for statistical comparisons in biomechanical characterization studies as provided here are critical to acknowledge potential sex-dependent differences in both physical and computational human head models in the future. This may help to overcome the sex bias towards men in tissue mechanics and its respective application fields^44^. Lastly, the independence of the here investigated mechanical parameters from PMI within a median of 70 h indicates that human neurocranium samples have consistent failure characteristics within this given time frame, and, therefore, can be used for biomechanical purposes as well as grafts in transplant surgery when kept at a maximum temperature of 4 °C.

### Limitations

The study is limited in sample size as the here given number of fresh tissues was the maximum that could be allocated to this project. The neurocranial samples that were used for the three-point bending tests in this study represent an inhomogeneous, anisotropic material with a varying cross-section along their lengths. Therefore, it is an oversimplification to assume a straight beam with a rectangular surface for B_strength_ calculations. As the human neurocranial bone forms an anisotropic material^45,46^ the here obtained mechanical parameters are only valid for the applied loading axis, which was chosen in order to reflect the extracranial–intracranial loading axis in vivo to the best possible extent.

## Conclusions

The fracture loads of human neurocranial samples increase with increasing sample thickness and testing velocities between 2.5 and 3.5 m/s in a three-point bending setup. The B_strength_ of neurocranial samples decreases depending on age at death and the thickness of the tested sample. The fracture loads of the human neurocranium are sex-independent, but sex-dependence of B_strength_ warrants further investigation. The here investigated biomechanical parameters of human neurocranial bone remained constant over a median time frame of 70 h post mortem when being cooled at 4 °C.
